# Evaluation of Oral Health-Related Quality of Life Following Laser Gingival Depigmentation: A Metric Questionnaire-Based Observational Study

**DOI:** 10.7759/cureus.90504

**Published:** 2025-08-19

**Authors:** Khyati Arora, Harikumar Kanakkath, Sanara PP, Mohammed Shereef, Prashansa Sharma

**Affiliations:** 1 Periodontology and Implantology, Government Dental College, Kozhikode, Kerala University of Health Sciences (KUHS) Thrissur, Kozhikode, IND; 2 Periodontology and Implantology, ITS Dental College, Muradnagar, IND

**Keywords:** facial aesthetics, gingiva, laser depigmentation, oral health, quality of life

## Abstract

Introduction: Gingival hyperpigmentation, often caused by excessive melanin deposition, can compromise smile aesthetics and affect an individual's self-esteem. As aesthetic demands in dentistry continue to rise, gingival depigmentation has emerged as a patient-driven solution to enhance oral appearance. This study investigates the effect of laser-assisted gingival depigmentation on oral health-related quality of life (OHRQoL) in young adults, using a validated metric questionnaire.

Methods: Young patients with varying degrees of gingival pigmentation underwent laser depigmentation with a laser device, and OHRQoL scores were assessed pre- and post-treatment using a detailed questionnaire designed for young adults. Statistical analysis was conducted to determine the significance of improvements across psychosocial and functional domains.

Results: Laser-assisted gingival depigmentation significantly improved oral health-related quality of life across all domains. The total OHRQoL score decreased from a median of 28.8 ± 8.72 before treatment to 13.77 ± 1.88 after treatment (Z = -5.162, p < 0.001). Improvements were observed in social function (paired difference = 3.80 ± 2.79), physical function (paired difference = 3.17 ± 1.97), and self-perception/anxiety (paired difference = 8.06 ± 3.02). Participants with a Dummett-Gupta score of 3 showed a greater reduction (paired difference = 17.41 ± 7.75) compared to those with a score of 2 (paired difference = 11.00 ± 5.81), indicating enhanced aesthetic and psychosocial benefits in individuals with higher baseline pigmentation.

Conclusion: Laser gingival depigmentation significantly improves OHRQoL by enhancing patient confidence, social interactions, and functional well-being. These results underscore its role as a minimally invasive and effective approach in periodontal aesthetic treatments. Future research should explore long-term outcomes and standardized psychological assessment methods for a more comprehensive understanding of patient-reported benefits.

## Introduction

A beautiful smile surely enhances an individual's self-confidence. The harmony of the smile is attributable to the shape, color, and position of the teeth in conjunction with the gingival tissue [1]. Gingival tissue constitutes the macroelement of dentofacial aesthetics along with the face, lip, and teeth [2]. Hyperpigmented gingiva has a key impact on facial aesthetics, thus affecting the oral health-related quality of life (OHRQoL). Gingival health and appearance are essential components for an attractive smile, and removal of unsightly pigmented gingiva is necessary for a pleasant and confident smile [3].

Gingival color is generally described as "coral pink." Excessive deposition of melanin located in the basal and suprabasal cell layers of the epithelium will result in gingival hyperpigmentation [4]. Gingival hyperpigmentation can be defined as a darker gingival color beyond what is normally expected [5]. The pigmentation may also occur as a consequence of benign and malignant lesions, intentional tattooing, drugs, heavy metal ingestions, smoking, systemic diseases, conditions, etc. [6,7]. Ethnicity and age also influence the color of the gingiva and have no sexual predilection [8]. Gingival depigmentation can be defined as a periodontal plastic surgical procedure whereby the gingival hyperpigmentation is removed or reduced by various techniques [9]. Depigmentation is not a clinical indication but a treatment of choice where aesthetics is a concern and is desired by the patients [3,10]. Gingival hyperpigmentation is removed or reduced by using different techniques of gingival depigmentation. The traditional dentist-oriented focus was mainly on the biological aspects.

Although this has not been abandoned, modern dentistry is more patient-oriented, and today, aesthetics is considered an important and integral part of an individual's oral health. Oral health is defined as health associated with the mouth, which includes the mouth, teeth, gums, supporting tissues, and branches of the nervous, immune, and vascular systems [11]. From a conceptual perspective, the move toward metrics evaluating OHRQoL is extremely relevant to dental treatment, to restore function, aesthetics, and comfort for the patient. Gingival depigmentation improves facial aesthetics, which in turn should improve oral health-related quality of life. This study assessed the impact of laser-assisted gingival depigmentation on oral health-related quality of life in young adults, using a targeted metric questionnaire developed by Daneshvar et al. [12].

## Materials and methods

Study design

This was a questionnaire-based observational study.

Study setting

The study was conducted in the Department of Periodontics at Government Dental College, Kozhikode. The study population consisted of patients visiting the outpatient department for gingival depigmentation.

Inclusion and exclusion criteria

We included adult patients aged between 21 and 35 years with mild to heavy pigmentation, classified using the Dummett-Gupta Oral Pigmentation Index [13]. Those who intended to undergo depigmentation were included. Patients who could not understand English, were smokers, had mal-aligned teeth, exhibited extrinsic or intrinsic stains, had caries or restorations in the anterior tooth region, were pregnant or lactating, or were under medication for psychiatric illnesses or systemic diseases were excluded from the study.

**Figure 1:**
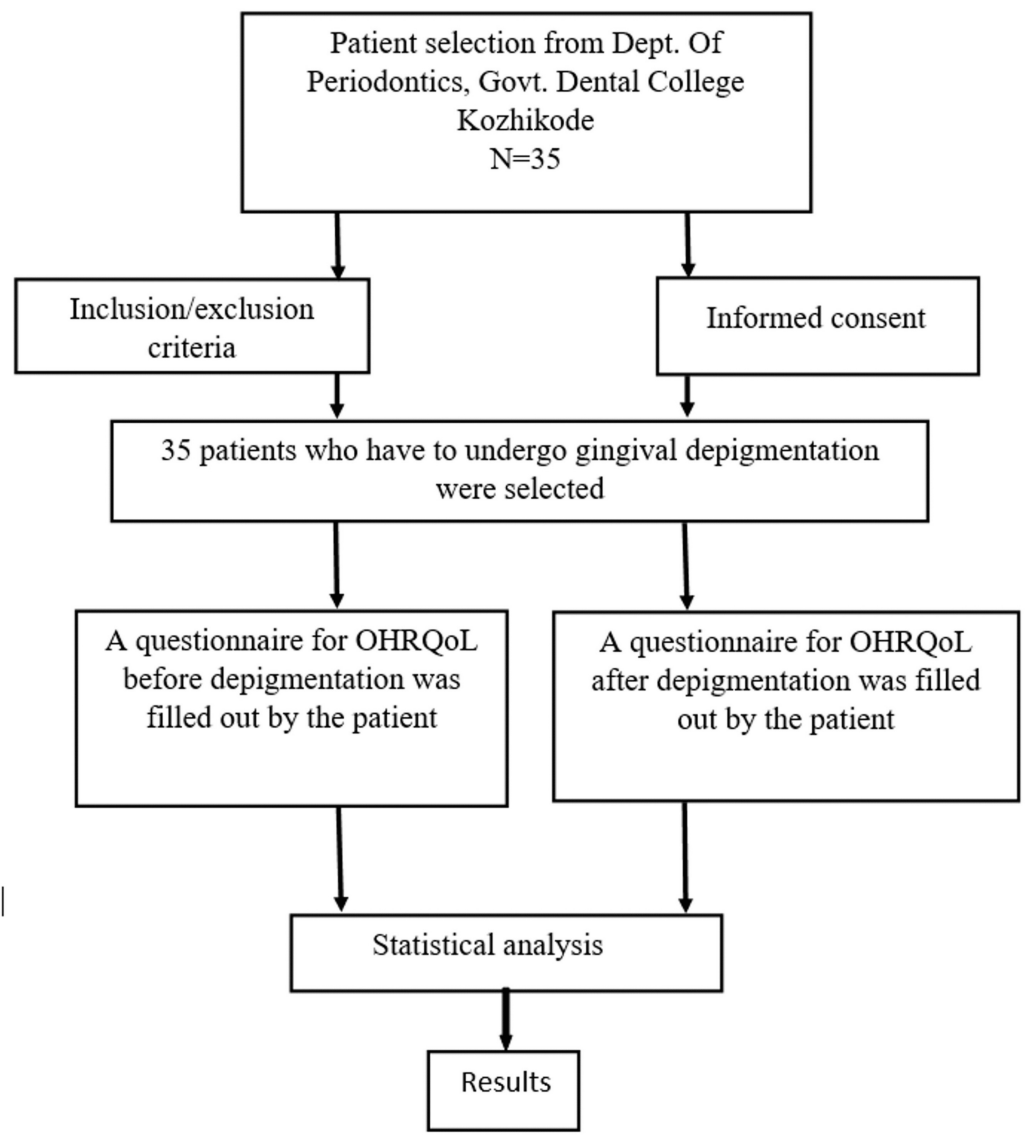
Flowchart of the study participant selection process OHRQoL: oral health-related quality of life

The study participant selection process is shown in Figure 1.

Study duration

The study was conducted over six months. It commenced on 14/02/2024 and concluded on 14/08/2024. The Institutional Ethics Committee of Government Dental College, Kozhikode issued approval 267/2023/DCC dated 18/05/2023. The study was prospectively registered in the Clinical Trial Registry of India (CTRI/2024/02/062453 dated 08/02/2024).

Study procedure

**Figure 2:**
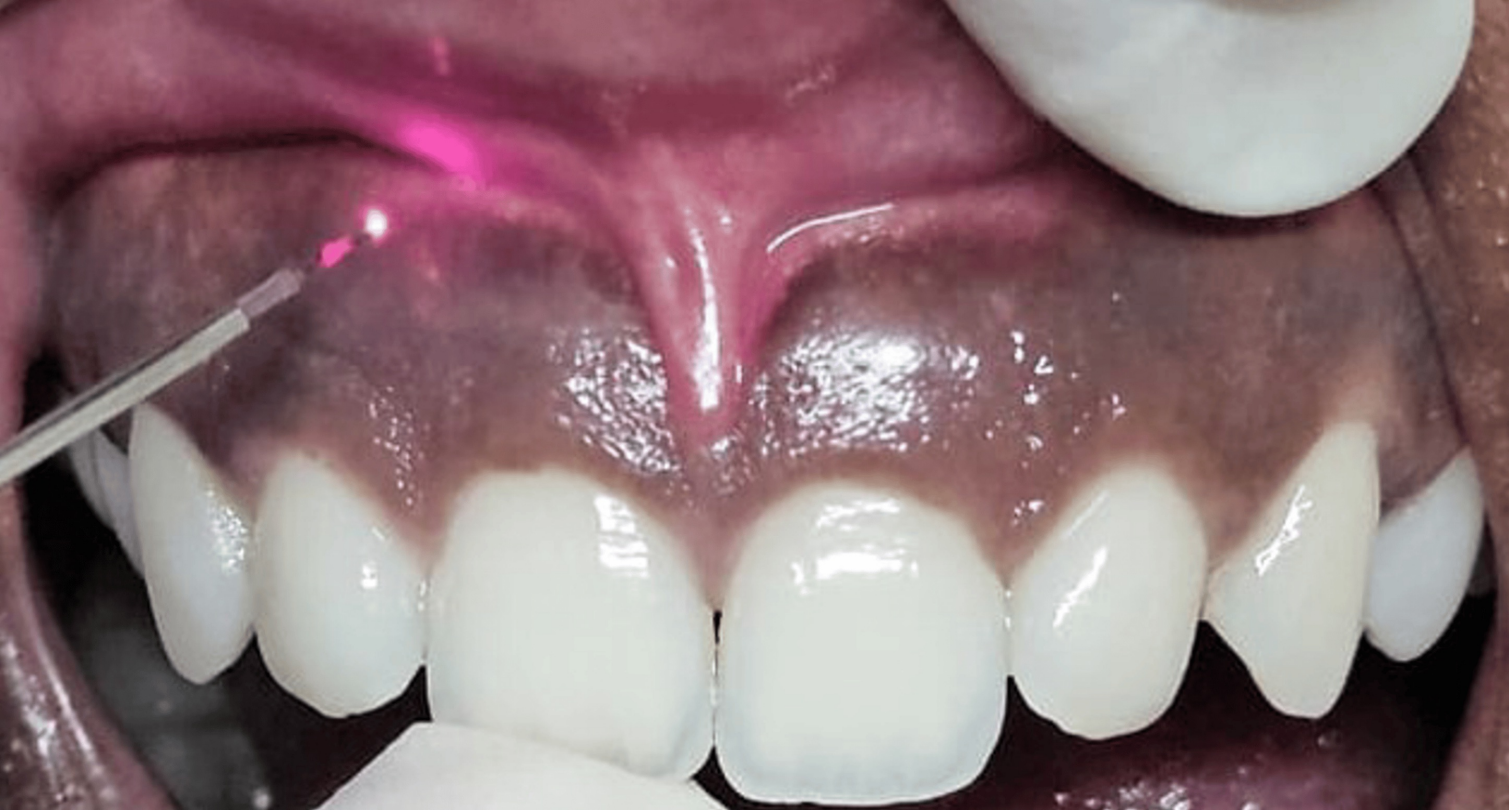
Laser irradiation

Patients who underwent laser gingival depigmentation were included in the study. A single trained periodontist performed laser irradiation on all patients using fixed power and energy settings with a fiber optic pliable tip (Figure 2). Irradiation was done using a diode laser (Biolase ezlase 940 Dental Laser; BIOLASE, Inc., Foothill Ranch, CA) with the following settings: wavelength of 940 nm, power of 1.5 W in a continuous mode, and energy of 151 J for three minutes per site from canine to canine, with total time ranging from 15 to 20 minutes. The pigmented area was divided into vertical rows, irradiated from one side to another. The laser was applied in contact mode and at a 45-degree angle to the tissue, avoiding damage to the neighboring teeth. The brushing motion was focused on pigmented spots, moving from the attached gingiva toward the free gingiva without needing an air cooling system until blister formation raised in the gingiva. It was scraped off with the sterile gauze soaked in saline water to remove the pigmented epithelium.

**Figure 3:**
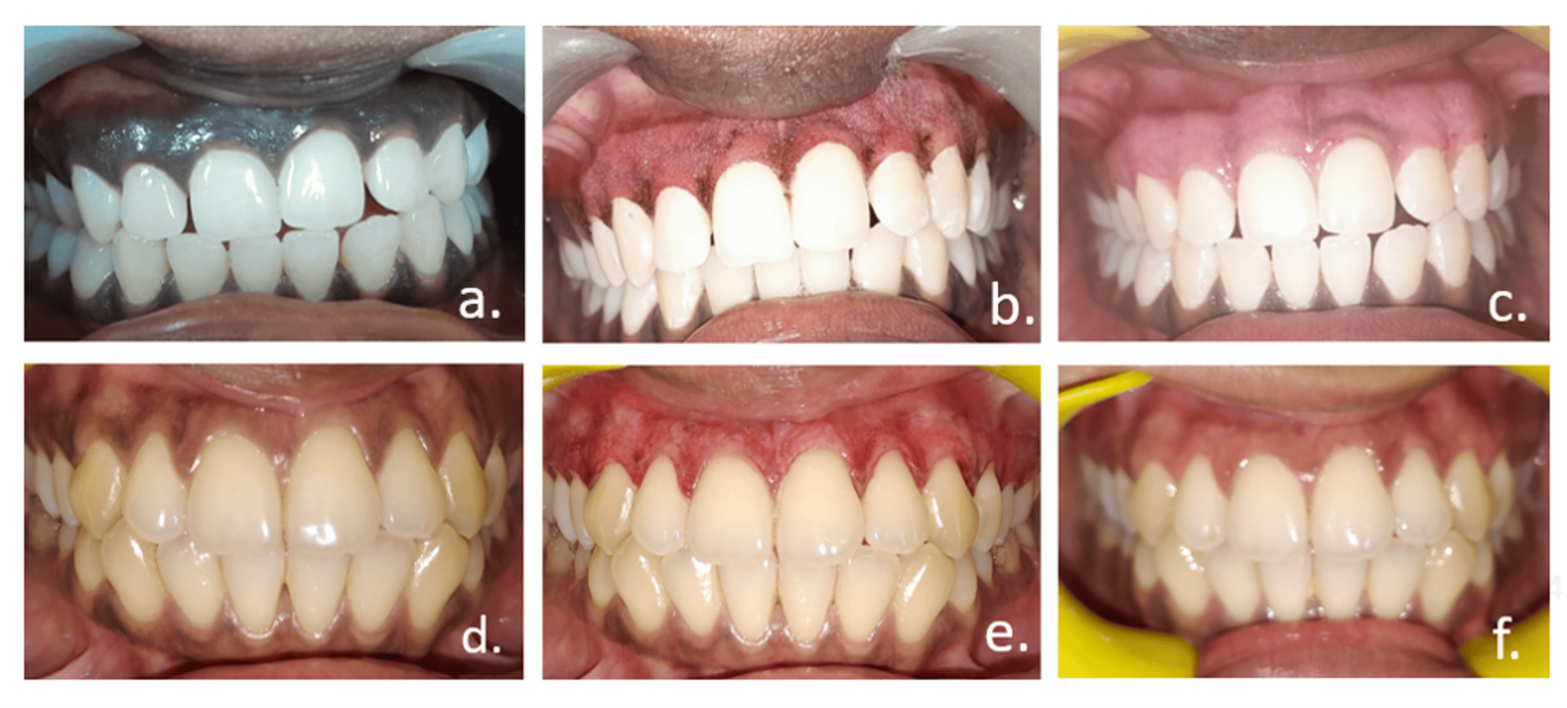
Laser gingival depigmentation: evaluating aesthetic and clinical outcomes after one-month follow-up a: preoperative (DS3), b: intraoperative (DS3), c: one-month follow-up (DS3), d: preoperative (DS2), e: intraoperative (DS2), f: one-month follow-up (DS2) Dummett-Gupta Oral Pigmentation Index [13]: DS0: Dummett-Gupta score of 0, DS1: Dummett-Gupta score of 1, DS2: Dummett-Gupta score of 2, DS3: Dummett-Gupta score of 3

Each participant completed a detailed questionnaire covering personal information, sociodemographic characteristics, medical history, drug history, and oral hygiene practices. Additionally, the oral health-related quality of life (OHRQoL) questionnaire [12] was administered both before the depigmentation procedure and one month afterward to assess changes in patient perception and functional outcomes. Two questionnaires were utilized to quantify the impact: one evaluating OHRQoL before the intervention and the other assessing it post-treatment. The collected responses were systematically scored, and the data were analyzed using appropriate statistical methods to identify significant differences and trends in patient-reported outcomes. Figure 3 provides an illustration of aesthetic and clinical outcomes.

Plan of analysis

Data management and analysis were conducted using SPSS version 23 (IBM Corp., Armonk, NY). The significance level (α) was set at 5%, with a power (1-β) of 80%. Test of normality showed that data were not normally distributed; therefore, the Wilcoxon signed-rank test was used to compare social function, physical function, self-perception, and anxiety before and after laser gingival depigmentation in all study participants.

## Results

The present study analyzed a cohort of 35 participants, with a mean age of 24.23 ± 6.08 years. The demographic profile revealed a predominance of female participants, most of whom were students from above poverty line (APL) households. Educational attainment varied, although the majority had completed high school or held a graduate degree. Baseline pigmentation analysis using the Dummett-Gupta Oral Pigmentation Index indicated that a significant proportion of participants exhibited severe pigmentation, with a greater number falling under a score of 3. Detailed demographic characteristics are presented in Table 1.

**Table 1:** Demographics of the study participants (N = 35) APL: above poverty line, BPL: below poverty line

Variables		Number (%)
Gender	Male	8 (22.9)
	Female	27 (77.1)
Religion	Hindu	14 (40.0)
	Muslim	16 (45.7)
	Christian	5 (14.3)
Education	Middle school	3 (8.60)
	High school	14 (40.0)
	Diploma	1 (2.9)
	Graduate	16 (45.7)
	Professional	1 (2.9)
Occupation	Unemployed	3 (8.6)
	Unskilled	1 (2.9)
	Semi-skilled	2 (5.7)
	Skilled	2 (5.7)
	Semi-professional	4 (11.4)
	Professional	1 (2.9)
	Students	22 (62.9)
Sociodemographic	APL	19 (54.3)
	BPL	16 (45.7)
Dummett-Gupta score	2	13 (37.1)
	3	22 (62.9)

Post-treatment assessment demonstrated significant improvement across all domains of oral health-related quality of life (OHRQoL). Self-perception scores displayed the most notable enhancement, indicating a substantial shift in participants' satisfaction with their oral appearance. Social function and physical function domains also improved markedly, suggesting enhanced confidence and reduced discomfort in interpersonal and functional settings. Anxiety scores showed a meaningful decrease, reflecting psychological relief and reduced self-consciousness regarding gingival aesthetics. These outcomes are presented comprehensively in Table 2.

**Table 2:** Wilcoxon signed-rank test statistics comparing social function, physical function, self-perception, and anxiety before and after laser gingival depigmentation in all study participants *p < 0.05 IQR: interquartile range, Z: approximation test statistics

Study participants (N = 35)	Before laser gingival depigmentation (median ± IQR)	After laser gingival depigmentation (median ± IQR)	Paired difference	Z	p-value
Social function	9.40 ± 3.76	5.60 ± 0.97	3.80 ± 2.79	-4.555	<0.001*
Physical function	6.51 ± 2.51	3.34 ± 0.54	3.17 ± 1.97	-4.646	<0.001*
Self-perception and anxiety	12.89 ± 4.22	4.83 ± 1.20	8.06 ± 3.02	-5.020	<0.001*
Total	28.8 ± 8.72	13.77 ± 1.88	15.03 ± 6.84	-5.162	<0.001*

When examined according to pigmentation severity, individuals with a Dummett-Gupta score of 3 consistently reported higher baseline impairment across OHRQoL domains. Following laser treatment, these participants demonstrated more substantial improvements in self-perception, social integration, physical comfort, and anxiety relief compared to those with a Dummett-Gupta score of 2. This pattern suggests that individuals with more pronounced pigmentation derive greater benefit from the intervention. Comparative data and subgroup outcomes are detailed in Table 3.

**Table 3:** Wilcoxon signed-rank test statistics comparing social function, physical function, self-perception, and anxiety before and after laser gingival depigmentation with Dummett-Gupta scores 2 and 3 *p < 0.05 IQR: interquartile range, Z: approximation test statistics

Study participants (N = 35)		Before laser gingival depigmentation (median ± IQR)	After laser gingival depigmentation (median ± IQR)	Paired difference	Z	p-value
Dummett-Gupta score 2 (n = 13)	Social function	7.31 ± 3.01	5.23 ± 0.44	2.07 ± 2.69	-2.207	0.027*
	Physical function	5.69 ± 1.79	3.31 ± 0.48	2.38 ± 1.71	-2.820	0.005*
	Self-perception and anxiety	11.00 ± 2.67	4.46 ± 1.13	6.54 ± 2.50	-3.189	0.001*
	Total	24.00 ± 6.12	13.00 ± 1.47	11.00 ± 5.81	-3.184	0.001*
Dummett-Gupta score 3 (n = 22)	Social function	10.64 ± 3.66	5.82 ± 1.14	4.82 ± 2.90	-4.030	<0.001*
	Physical function	7.00 ± 2.77	3.36 ± 0.58	3.64 ± 2.42	-3.746	<0.001*
	Self-perception and anxiety	14.00 ± 4.61	5.05 ± 1.05	8.95 ± 4.07	-3.931	<0.001*
	Total	31.63 ± 8.88	14.22 ± 1.97	17.41 ± 7.75	-4.109	<0.001*

Overall, the results indicate that laser gingival depigmentation significantly enhances both psychological and functional aspects of oral health-related quality of life. The improvement trend was consistent regardless of gender, age, or socioeconomic background, underscoring the universal value of this aesthetic intervention. Statistical significance across OHRQoL domains was confirmed and is summarized in Table 3.

## Discussion

A healthy smile is achieved by the shape of the tooth and the appearance of the gingiva. A healthy gingiva plays an important role in a delightful smile. In our daily clinical practice, gingival depigmentation is a regular demand, and it is usually for aesthetic reasons. The application of laser technology in gingival depigmentation offers several advantages over conventional methods, such as scalpel surgery, cryotherapy, and chemical methods. The diode laser exhibits the "hot tip" effect, which results in heat accretion at the end of the fiber, forming a coagulation layer on the surface treated. The usual mechanisms of a diode laser are photochemical, thermal, or plasma-mediated [14]. With the temperature rise, the soft tissues are exposed to warming (37°C-60°C), protein denaturation, coagulation (>60°C), welding (70°C-90°C), vaporization (100°C-150°C), and finally carburization (>200°C) [15]. The 940 nm diode laser was used as its absorption is high in hemoglobin and other pigments. In addition, the diode laser causes minimal destruction to the periosteum and bone. The added advantage of the diode laser is its bactericidal effect, thereby producing a sterile condition concomitant with the operation, as shown in an in vivo and in vitro study by Patel et al. [16]. Lasers provide precise tissue ablation, minimal discomfort, faster healing, and reduced postoperative complications, making them an ideal choice for soft tissue procedures [17].

Tooth color and the visibility of gums correlated with satisfaction with the smile [18]. The fact that tooth color is one of the most important factors in satisfaction with oral appearance is in accordance with the self-perception study of Neumann et al. [19]. Previous studies have highlighted the impact of gingival pigmentation on aesthetics and self-confidence, with excessive pigmentation often being a concern for patients seeking dental treatment [20]. Furthermore, the improvements in social interaction metrics support the premise that aesthetic dental treatments contribute to better psychosocial outcomes [21]. The findings of this study demonstrate a significant improvement in oral health-related quality of life (OHRQoL) following laser gingival depigmentation. The reduction in social function, physical function, and self-perception scores post-treatment suggests that the procedure positively influences patient well-being.

The novelty of this study lies in its prospective design and focus on young adults, a demographic often underrepresented in OHRQoL research. To date, no longitudinal studies have systematically evaluated the psychosocial outcomes of laser depigmentation in this age group, making the present findings particularly relevant for clinicians aiming to adopt patient-centered approaches in periodontal aesthetics. The statistically significant differences in OHRQoL scores, particularly among patients with higher Dummett-Gupta pigmentation scores, reinforce the effectiveness of laser depigmentation in enhancing psychological and functional aspects of oral health.

The clinical implication of this study lies in its successful quantification of patient perception using a structured and metric-based format. This approach enables clinicians to objectively assess subjective experiences, thereby facilitating more patient-centered decision-making and tailored treatment strategies.

However, certain limitations must be acknowledged. The relatively small sample size and short follow-up duration (one month) limit the generalizability and long-term applicability of the results. While no recurrence was observed within the follow-up period, melanin repigmentation is a known phenomenon influenced by factors such as melanocyte migration and genetic predisposition. Physiological recurrence of the pigmentation has been reported usually after more than six months. An acceptable explanation for this is the migration theory described by Perlmutter and Tal [22]. Future studies should incorporate extended follow-up intervals (6-12 months or longer) to assess recurrence rates, patient satisfaction, and sustained improvements in OHRQoL.

Additionally, incorporating histological assessments and patient-reported outcome measures (PROMs) could provide a more comprehensive understanding of tissue response and subjective satisfaction. Comparative studies evaluating different laser wavelengths, power settings, and treatment protocols would also help establish standardized guidelines for optimal clinical outcomes.

## Conclusions

The findings of this study affirm that laser-assisted gingival depigmentation significantly enhances oral health-related quality of life (OHRQoL), particularly in domains related to self-perception, social confidence, and functional well-being. By addressing the aesthetic concerns associated with gingival hyperpigmentation, the procedure contributes not only to improved smile aesthetics but also to measurable psychosocial benefits, especially among young adults, a demographic often sensitive to appearance-related self-esteem. The use of diode lasers offers a minimally invasive, patient-friendly alternative to conventional depigmentation techniques, with advantages such as precise tissue ablation, minimal intraoperative bleeding, reduced postoperative discomfort, and accelerated healing. These attributes make laser therapy a compelling option for integration into routine periodontal practice, particularly in cases where aesthetic outcomes are a primary concern. Moreover, the study highlights the importance of incorporating patient-centered outcomes, such as OHRQoL metrics, into the evaluation of periodontal interventions. This shift toward holistic assessment underscores the evolving role of periodontics in enhancing not only oral health but also overall quality of life. In summary, laser gingival depigmentation emerges as a safe, effective, and psychologically beneficial intervention that aligns with modern principles of minimally invasive and patient-centered dental care.

## Appendices

Table 4 shows the oral health-related quality of life questionnaire used in the present study.

**Table 4:** Oral health-related quality of life questionnaire The study utilized the oral health-related quality of life questionnaire from the "Oral health related quality of life: a novel metric targeted to young adults" developed by Daneshvar et al. [12], with formal permission obtained via email from the author.

Questions		Responses
a. Social function	1. How often have you felt that problems with your teeth or mouth have affected your friendships?	1. Never, 2. Once, 3. More than once, 4. Often, 5. Very often
	2. How often have you felt that problems with your teeth and mouth have affected your work?	1. Never, 2. Once, 3. More than once, 4. Often, 5. Very often
	3. How often have you avoided contact with other people because of the way your teeth and mouth look?	1. Never, 2. Once, 3. More than once, 4. Often, 5. Very often
	4. How often have you avoided eating with others because of your teeth and mouth?	1. Never, 2. Once, 3. More than once, 4. Often, 5. Very often
	5. How often have you felt that your teeth and mouth have affected your romantic life?	1. Never, 2. Once, 3. More than once, 4. Often, 5. Very often
b. Physical function	6. How often has your focus been affected because of problems with your teeth and gums?	1. Never, 2. Once, 3. More than once, 4. Often, 5. Very often
	7. How often have you missed school or work because of problems with your teeth or mouth?	1. Never, 2. Once, 3. More than once, 4. Often, 5. Very often
	8. How often have you felt that life, in general, was less enjoyable because of problems with your teeth or mouth?	1. Never, 2. Once, 3. More than once, 4. Often, 5. Very often
c. Self-perception and anxiety	9. How often have you been self-conscious because of your teeth or mouth?	1. Never, 2. Once, 3. More than once, 4. Often, 5. Very often
	10. How often have you felt that problems with your teeth or mouth have affected your appearance?	1. Never, 2. Once, 3. More than once, 4. Often, 5. Very often
	11. How often have you been worried by problems with your teeth or mouth?	1. Never, 2. Once, 3. More than once, 4. Often, 5. Very often
	12. How often have you avoided smiling or laughing because of problems with your teeth or mouth?	1. Never, 2. Once, 3. More than once, 4. Often, 5. Very often
